# Gum Arabic modulates the microbiota-gut-brain axis and affects general fitness in zebrafish

**DOI:** 10.1038/s41598-025-17665-z

**Published:** 2025-10-02

**Authors:** Justin Abi Assaf, Jean-Charles de Coriolis, Alice May Godden, Eve Redhead, Jamie Bartram, Jayme Cohen-Krais, Karina Silova, Zoe Crighton, Gwenaelle Le Gall, Saber Sami, Sami Ahmed Khalid, Simone Immler

**Affiliations:** 1 School of Biological Sciences, University of East Anglia, Norwich Research Park, Norwich, NR4 7TJ UK; 2 Norwich Medical School, University of East Anglia, Norwich Research Park, Norwich, NR4 7TJ UK; 3 Faculty of Pharmacy, University of Science and Technology, Omdurman, Sudan

**Keywords:** Dietary fibre, Reproductive fitness, Locomotion, Brain transcriptome, Acetate, *Cetobacterium*, Metabolomics, RNA, Bioinformatics, Experimental organisms, Computational biology and bioinformatics, Ecology, Microbiology, Molecular biology, Physiology, Systems biology

## Abstract

Gum Arabic (GA) (*Acacia senegal* var. *senegal*) is an edible tree exudate and dietary fibre shown to improve health in humans and animals. We tested the ramifications of GAon organismal health across the microbiota-gut-brain axis by supplementing female and male zebrafish (*Danio rerio*) with two concentrations(6% or 60%) of GA (*Acacia senegal* var. *senegal*) for two weeks. We assessed the effects on the gut microbiome composition, intestinal and brain metabolic profiles, reproductive fitness, locomotion, and brain gene expression. GA supplementation induced a relative decrease in Proteobacteria and a relative increase in Fusobacteria, with a rise in the beneficial genus *Cetobacterium*. In the GA-supplemented fish, we detected increased intestinal glucose metabolism, evidenced by reduced glucose retention levels. Additionally, high levels of acetate were detected in the brain. Interestingly, the gene *cart1*, involved in appetite and hunger control, was significantly downregulated in female brains only. Consistently, we detected increased locomotion in GA-supplemented fish compared to Control fish. Interestingly, GA supplementation had a negative effect on female reproductive fitness and a positive effect on male reproductive fitness. .Our results emphasise the significance of evaluating the impact of dietary fibre at a systemic level to develop relevant nutritional guidelines that consider the different nutritional requirements of each sex.

**Supplementary Information:**

The online version contains supplementary material available at 10.1038/s41598-025-17665-z.

## Introduction

Diet modulates the dynamic nature of the gut microbiome, influencing not only its composition but also its function and diversity. Different diets vary in their nutritional profile, whole-food composition, caloric value, and dietary fibre (DF) content, all of which can alter the microbial architecture, leading to either positive or negative health ramifications^1^. A diet rich in DFs is essential for optimal health and well-being by preventing various non-communicable diseases due to its putative positive effects on cardiovascular, metabolic, and cognitive health in addition to weight management, appetite regulation, and inflammation^1–5^. DFs constitute a wide range of heterogeneous compounds that resist digestion, and the gut microbiome plays a key role in breaking them down to expose their beneficial properties to the host organism^6,7^. This bidirectional interaction between the gut microbiome and DFs not only influences intestinal health and metabolism but also mediates host behaviour and fitness via the gut-brain axis^8,9^.

Gum Arabic (GA) is an edible tree gum exudate obtained by the incision of the trunks and the branches of *Acacia senegal* (L.) Willdenow, native to the arid and semi-arid regions of Africa. Also known by several names like gum Acacia, Senegal gum, and Sudan Arabic gum, GA is chemically composed of highly branched and high molecular weight glycoproteins and polysaccharide complexes of which 90% is arabinogalactan, a polymer of arabinose (17–34%) and galactose (32–50%) with prebiotic properties, in addition to some amino acids and minerals^10,11^. It is a key additive (E-Number 414) in the food industry, mainly used as an emulsifier and a stabiliser, but its potential benefits as a DF have been largely overlooked^12^. Traditional medicine has long suggested health benefits from its oral consumption, particularly with reports indicating positive effects on vital organ functions, such as the kidneys^13^, the brain^14^, the heart^15^, and the liver^16^ in both humans and animals. Although not primarily consumed in the Western world as a source of DF, people in sub-Saharan regions like Sudan have incorporated GA into their day-to-day life for both medicinal and alimentary purposes long before it was considered safe for human consumption by regulatory bodies like the Food and Drug Administration (FDA) and the Food and Agriculture Organization (FAO) in the 1970s^17–19^.

GA displays a range of potential health benefits. A randomised clinical trial revealed that after 12 weeks of receiving 20 g/day of GA, participants showed a significant decrease in systolic and diastolic blood pressure and fasting blood glucose^20^. Similarly, the administration of GA was associated with improved renal function in patients with progressive chronic kidney disease (CKD)^13^, and the oral administration of 30 g GA per day had antioxidant and anti-inflammatory effects in patients undergoing haemodialysis^21^. A recently published systematic review of 29 clinical trials utilising GA as a therapeutic agent for various human diseases and conditions indicated that GA improves oral health, gastrointestinal functions, and supports healthy weight management in addition to alleviating the adverse effects of sickle cell anaemia and rheumatoid arthritis^22^. Beneficial effects have also been identified in mice where the administration of GA mitigated the expression of key pro-inflammatory plasma cytokines: IL-6, IL-1β, and TNF-α; despite being fed an inflammatory high-fat diet^23^. Furthermore, GA improved semen quality in rats^24^ and ovarian function in mice^25^ while exhibiting no teratogenic effects^26^; and in rats, GA enhanced cognitive ability whilst demonstrating neuroprotective properties^14,27^. Additionally, GA has prebiotic and modulatory effects on the gut microbiome^10,28–30^ via its fermentation by bacteria into microbial-derived metabolites, mainly short-chain fatty acids (SCFAs)^29,31,32^. This microbiota-gut-brain axis is directly influenced by the diet, which in turn can affect the cognitive behaviour and immunity of the organism^33–35^. The broad range of health benefits associated with GA supplementation suggests that its effects are likely to occur at various levels along the gut-brain axis; however, the exact mechanisms are not yet fully understood.

**Fig. 1:**
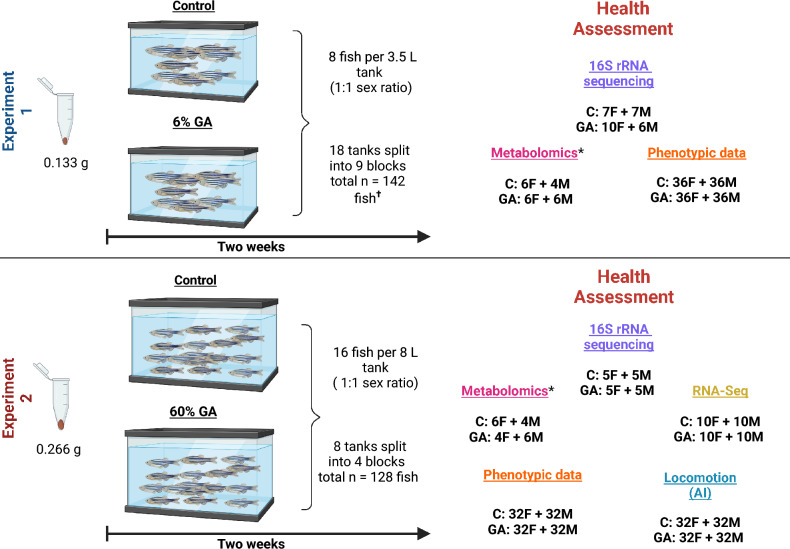
Overview of the experimental design for both experiments using 6% and 60% GA* supplementation. The total number and sex (F: female, M: male) of the fish used in the two independent experiments and the sample size for each health assessment are shown. ^†^Two fish died, ^*^Controls were pooled from both experiments. The figure was created in BioRender.com.

The nutritional requirements of DFs in zebrafish (*Danio rerio*) are underexplored and under-characterised, with few studies linking DF supplementation to whole-organism phenotypes. In this study, we assessed the health implications of *Acacia senegal* var. *senegal* (hereafter referred to as GA*) supplementation at multiple levels, from the gut microbiome to brain function, including reproductive behaviour, by experimentally manipulating the dietary supplementation of GA in zebrafish*.* Various human clinical trials^13,17^ have supported the health claims of GA consumption; however, these are not widely accepted in clinical practice due to the lack of mechanistic insight. We conducted a preclinical in vivo study using zebrafish, a non-mammalian vertebrate model, to elucidate and highlight both the benefits and potential trade-offs of dietary fibres, with particular focus on GA. We exposed female and male adult zebrafish (approximately 1 year old) to two GA-supplemented diets (6% or 60% GA*) for two weeks and compared them to Control fish fed a standard diet. No washout period was used. Immediately after the feeding period, we assessed reproductive fitness and locomotory behaviour, then collected the intestine and brain for gut microbiome profiling, tissue metabolomics, and brain transcriptomics (Fig. 1).

## Results

### The effects of GA on the gut microbiome

**Fig. 2:**
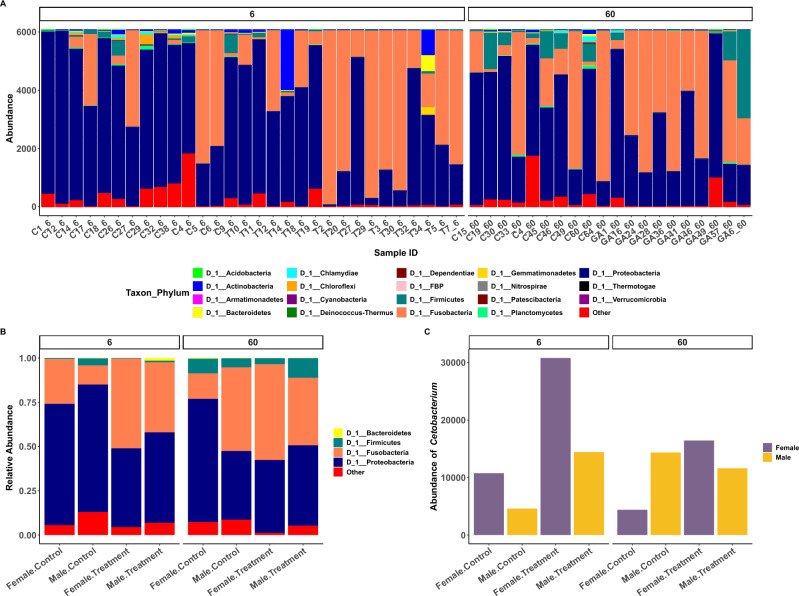
Microbiome composition stratified according to experimental conditions and sex. Abundance plot showing the two major phyla, Proteobacteria and Fusobacteria (**A**) in all zebrafish used in this analysis and (**B**) in the two sexes separately. We observed a general drop in the ratio of Proteobacteria/Fusobacteria in GA*-supplemented fish compared to Control. (**C**) Abundance of *Cetobacterium* in males and females, showing an increase in GA*-supplemented fish compared to Control fish, with a stronger effect in females.

To assess the effects of GA on the gut microbiome and its potential prebiotic properties, we performed 16S rRNA sequencing of the gut microbiome in fish with and without GA* supplementation. In line with previous findings, the dominant microbes in the zebrafish belong to two major phyla, Proteobacteria and Fusobacteria^36^ (Fig. 2A). GA* supplementation reduced the relative abundance of Proteobacteria in favour of an increase in Fusobacteria when compared to Control fish and the strength of the effect was similar in 6% and 60% GA* supplemented fish. However, the effects were stronger in females compared to males (Fig. 2B). The relative abundance of the main genus *Cetobacterium* in the Fusobacteria increased in females in both experiments whereas in males, we detected an increase only in the 6% GA* cohort (Fig. 2C and Fig. S2 in *SI Appendix*). This genus is of particular interest since it is beneficial for the health of various teleost species, including zebrafish, where it has been shown to enhance immunity against pathogens, to reduce inflammation, and to produce SCFAs^37–40^.

**Fig. 3:**
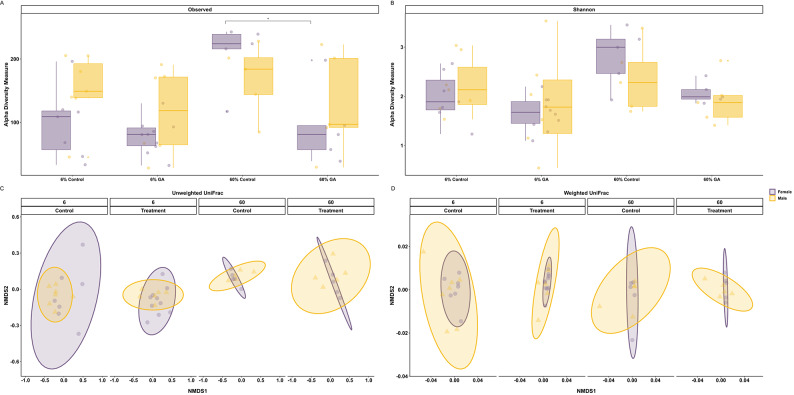
Microbial diversity assessed by α and β diversity. (**A**, **B**) Observed and Shannon indices of the gut microbiome based on 16S analysis. Under 6% GA*, no statistically significant changes in the α-diversity between the samples were detected. Under 60% GA*, only female fish displayed a significant drop (Kruskal–Wallis, * denotes *p *_adj_ = 0.003) in α-diversity compared to the Control indicated by the Observed index. Box plots represent the median, interquartile range, minimum, and maximum values with each dot representing a sample. (**C**) Unweighted UniFrac β-diversity based on 16S analysis, where 6% and 60% GA* supplementation resulted in a significant decrease, especially in females (PERMANOVA, *p* < 0.05). (**D**) Weighted UniFrac β-diversity based on 16S analysis where 6% GA* supplementation caused a decrease in both males and females (PERMANOVA, *p* < 0.05). However, we found no significant effects of 60% GA* supplementation.

We found no significant changes in α-diversity in either sex in the 6% GA* experiment as indicated by the Observed Operational Taxonomic Unit (OTU) richness and the Shannon diversity index (Fig. 3A and B). However, the OTU index (but not the Shannon index) indicated a decrease in microbial diversity when comparing female fish from the 60% GA* experiment to female Control fish (Dunn post-hoc after Kruskal–Wallis, Benjamini–Hochberg False Discovery Rate (FDR)^41^ *p *_adj_ = 0.003), but no such effect was apparent in males (Fig. 3A and B). This suggests that 60% GA* can potentially reduce the number of gut bacteria in females without affecting their relative abundance. In 6% GA*-supplemented fish, the unweighted and weighted UniFrac β-diversity were more uniform compared to Control fish as indicated by the tight clustering of samples and the narrow ellipses (Fig. 3C and D), and we found a significant effect of supplementation (PERMANOVA: *F* = 1.32, DF = 1, *p* = 0.043 and *F* = 3.3, DF = 1, *p* = 0.014) but no sex differences (PERMANOVA: *F* = 1.18, DF = 1, *p* = 0.1 and *F* = 2.24, DF = 1, *p* = 0.062), for unweighted and weighted UniFrac respectively. In fish under 60% GA* supplementation, both females and males showed changes in the microbial communities compared to Control fish as indicated by the unweighted UniFrac β-diversity index (Fig. 3C), as a direct effect of the 60% GA* dosage (PERMANOVA: *F* = 1.42, DF = 1, *p* = 0.021) but not sex (PERMANOVA: *F* = 1.02, DF = 1, *p* = 0.348). In contrast, the weighted UniFrac β-diversity index did not significantly differ between fish under 60% GA* and Control (Fig. 3D; PERMANOVA for treatment: *F* = 1.09, DF = 1, *p* = 0.357 and PERMANOVA for sex: *F* = 1.60, DF = 1, *p* = 0.17).

### The effects of GA on the metabolic profile of the intestine and the brain

**Fig. 4:**
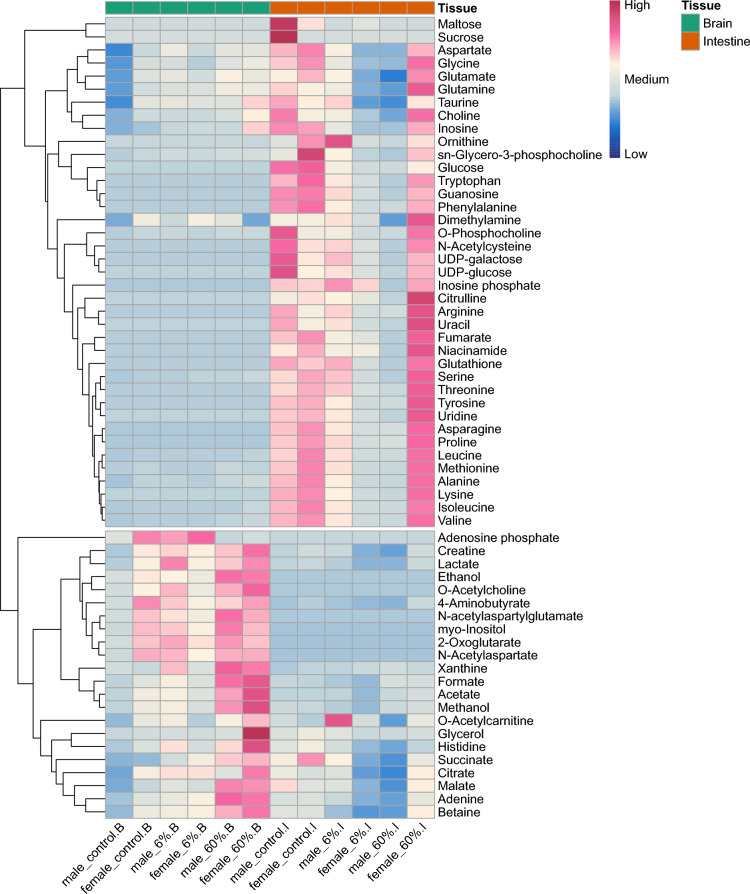
Heatmap displaying the 61 metabolites detected in the brain (B) and the intestine (I) by H^1^ NMR. The abundance of metabolites shows a tissue-specific pattern. Colours in pink indicate a relative increase and colours in blue indicate a relative decrease.

We performed ^1^H NMR (nuclear magnetic resonance) metabolic analysis to investigate the effects of GA supplementation on the metabolome, with a focus on the gut-brain axis. We identified and quantified 61 metabolites (*SI Appendix,* Table S2, Table S3, and Fig. S3) where 39 metabolites were detected in the intestinal samples and 22 metabolites were found in the brain (Fig. 4). Interestingly, acetate, one of the key SCFAs, was highly concentrated in the brain tissues of both female and male fish under 60% GA* supplementation compared to Control fish. In the brains of GA*-supplemented fish, we also observed a gradual increase in metabolites compared to the Control in both sexes, with most cerebral metabolites being detected in the 60% GA* fish. These detected brain and intestinal metabolites were most abundant in the 60% GA*-supplemented female fish.

### The effects of GA on organismal fitness

**Fig. 5:**
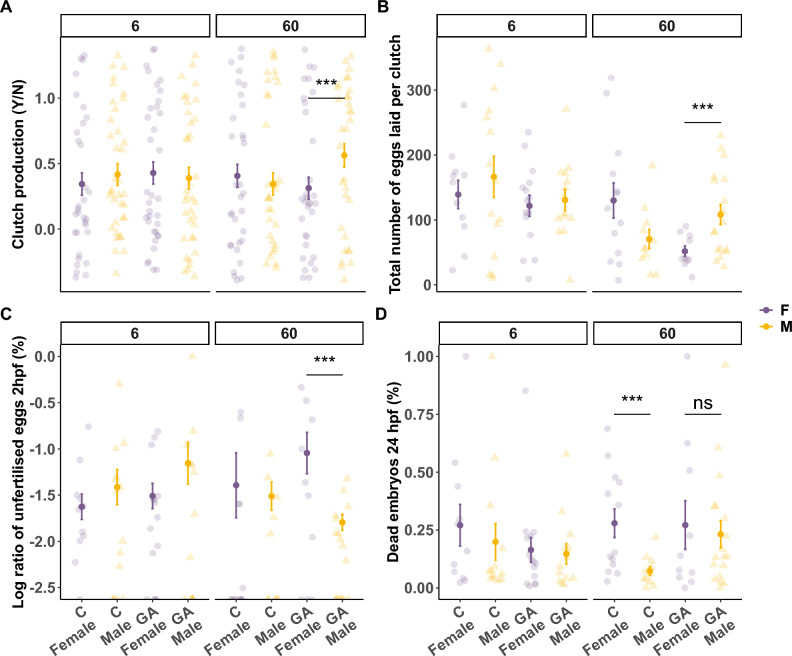
Scatter plots displaying measures of reproductive fitness obtained from spawning events between experimental and non-experimental fish. The centre bold dots and error bars indicate mean ± standard error and colours indicate males (purple) and females (yellow). (**A**) Clutch production during spawning (YES/NO) showed only a significant effect of GA under 60% GA* supplementation indicated by a significant interaction between Treatment:Sex. (**B**) The total number of eggs (clutch size) did not show any significant effects of 6% GA* supplementation, but we found a strong interaction effect between Treatment:Sex under 60% GA* supplementation, where GA*-supplemented females produced significantly fewer eggs per clutch compared to GA*-supplemented males. (**C**) Fertilisation success is measured as % unfertilised eggs. Data are plotted on a logarithmic scale for better visualisation. 6% GA* supplementation had no significant effects on the % of unfertilised eggs at 2 hpf, but 60% GA* supplemented females produced significantly more unfertilised eggs compared to Control females and more unfertilised eggs were detected when comparing 60% GA* females to the 60% GA* males. (**D**) Regarding embryo survival at 24 hpf, 60% GA* supplementation removed the sex-specific drop observed in the Control. The *** denote significance levels of comparisons based on statistical tests with *p* < 0.001, and ns signifies non-significance.

To test the effects of GA supplementation on organismal fitness, we assessed reproductive fitness in the experimental adult fish and their offspring (Fig. 5A–D). At the end of the two-week dietary intervention, both Control and GA* fish were crossed with non-experimental ABWT fish by natural spawning. By producing the eggs, female zebrafish have a direct physiological contribution to the clutch, whereas male zebrafish contribute indirectly by providing the sperm needed for egg fertilisation and by initiating the courtship and mating behaviour^42,43^. We found no statistically significant effects on clutch production (YES/NO) in fish under 6% GA* supplementation (binomial Generalised Linear Mixed-Effects Models (GLMER): $\upchi$^2^ = 0.40, *z* = 0.64, DF = 1, *p* = 0.52) nor 60% GA* supplementation (binomial GLMER: $\upchi$^2^ = 0.54, *z* = -0.73, DF = 1, *p* = 0.46) when compared to Control (Fig. 5A). Interestingly, the marginally non-significant interaction between 60% GA* and sex may indicate a subtle increase in reproductive success in males compared to females (binomial GLMER: $\upchi$^2^ = 3.18, DF = 1, *z* = 1.8, *p* = 0.075) (Fig. 5A). Similarly, neither 6% GA* (Poisson GLMER: $\upchi$^2^ = 0.10, *z* = -0.56, DF = 1, *p* = 0.76) nor 60% GA* supplementation (Poisson GLMER: $\upchi$^2^ = 0.13, *z* = -2.5, DF = 1, *p* = 0.72) had an impact on the total number of eggs produced (size of clutch) compared to the Control group (Fig. 5B). However, under 60% GA*, there was a significant interaction effect between treatment and sex (Poisson GLMER: $\upchi$^2^ = 12.56, *z* = 3.54, DF = 1, *p* < 0.001), indicating a sex-specific effect where GA*-supplemented females produced significantly smaller clutches compared to 60% GA* males (Poisson GLMER: Treatment:Sex interaction: *z* = − 3.11, DF = 1, *p* = 0.01) (Fig. 5B). Additionally, Control females produced significantly larger clutches compared to the 60% GA*-supplemented females (Poisson GLMER: Treatment:Sex interaction: *z* = 2.54, DF = 1, *p* = 0.05) (Fig. 5B).

Whereas 6% GA* supplementation had no significant effects on fertilisation success rate (binomial GLMER: $\upchi$^2^ = 0.08, *z* = -0.328, DF = 1, *p* = 0.774) (Fig. 5C) or embryo survival at 24 hpf (binomial GLMER: $\upchi$^2^ = 0.55, *z* = 1.5, DF = 1, *p* = 0.15) (Fig. 5D), 60% GA* supplementation resulted in a greater ratio of unfertilised eggs in females but not in males compared to Control fish (binomial GLMER: Treatment: $\upchi$^2^ = 4.52, *z* = -0.2, DF = 1, *p* = 0.03; Treatment:Sex interaction: $\upchi$^2^ = 2.92, *z* = -1.7, DF = 1, *p* = 0.09) (Fig. 5C). Also, 60% GA* females produced significantly more unfertilised embryos in comparison to the 60% GA* males (binomial GLMER: Treatment:Sex interaction: *z* = 2.7, DF = 1, *p* = 0.03) (Fig. 5C). Surprisingly, despite the 60% GA* males displaying better fertilisation rates, embryo survival at 24 hpf was no different than the ones derived from the crossing of 60% GA* females (binomial GLMER: Treatment:Sex interaction: *z* = 0.8, DF = 1, *p* = 0.86) (Fig. 5D). Refer to Table S4 in the *SI Appendix f*or the full statistical models. Overall, it appears that while GA* has some potentially beneficial effects on male reproductive fitness, the effects on female reproductive fitness, especially at high dosages, are detrimental.

**Fig. 6:**
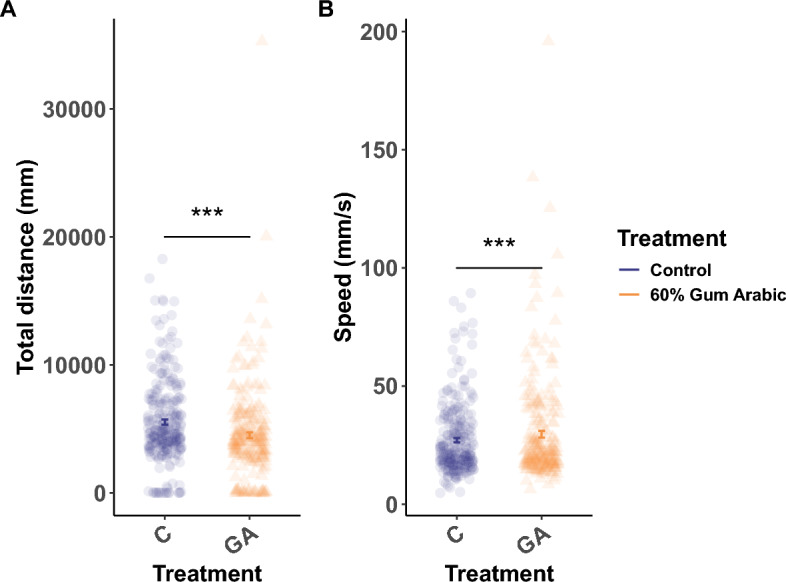
Scatter plots for locomotion showing total distance (A) and speed (B). The centre bold dots and error bars indicate the mean ± standard error. The *** denote significance levels of comparisons based on statistical tests with *p* < 0.001.

When assessing the activity of the adult fish by measuring locomotion using artificial intelligence of video recordings of shoaling fish, we focused on 60% GA* supplementation to associate this with the previously reported effects on reproductive fitness. We found that 60% GA*-supplemented fish covered a significantly shorter total distance (mm) compared to Control fish (Gaussian Linear Mixed-Effects Models (LMER): $\upchi$^2^= 30.49, *t* = -5.5, DF = 1, *p* < 0.0001) (Fig. 6A), but they swam faster (mm/s) (Gaussian LMER: $\upchi$^2^ = 15.53, *t* = 3.9, DF = 1, *p* < 0.0001) (Fig. 6B). This increase in activity in the GA*-supplemented fish could be a possible explanation for the contrasting effects on the reproductive fitness detected in females and males.

### The effects of GA on gene expression in the brain

Due to our observation of the substantial effects of 60% GA* supplementation on the gut microbiome and reproductive fitness, we performed whole transcriptome analysis on brain tissues in female and male fish under 60% GA* supplementation and tested for Differentially Expressed Genes (DEGs) (Table 1). When running a complete model with treatment and sex as factors, we found no DEGs when comparing 60% GA* to Control fish. However, when comparing gene expression in the brain between all the fish under 60% GA* supplementation and all Control fish without sex as a covariate, we found that *cart1* was downregulated, whereas *snora74* and *clec3ba* were upregulated in the 60% GA*-supplemented fish compared to the Control fish. When comparing Control females and Control males, we identified three statistically significant DEGs (*ier2a, egr1,* and *egr4*), that were upregulated in females compared to males, indicating a difference in brain function between the sexes. Two genes, *cart1* and *slc1a2A*, were significantly downregulated in 60% GA* females compared to Control females, but we found no DEGs when running the same analysis in males, further supporting a sex-specific response to GA* supplementation^44^. The gene *cart1* plays a role in feeding behaviour and appetite regulation^45^. The detected downregulation of the *cart1* gene in females suggests a sex-specific regulation of hunger in the brain. In this study, we employed a mixed-sex design (females and males housed together) and therefore both female and male fish were given the exact same amount of food (Control or 60% GA*). Although brain gene expression indicated sex-specific hunger regulation, neither females nor males fed 60% GA* showed statistically significant weight loss compared to sex-matched controls (lmer; *emmeans* contrasts, Tukey*p*_adj_ > 0.05) (Fig. S1 in the *SI Appendix*). Notably, the positive effect of GA* on brain function was supported by the upregulation of *clec3ba,* which is orthologous to human *CLEC3B* encoding for tetranectin, a protein with potential neuroprotective and immunomodulatory effects^46,47^.

**Table 1:** List of statistically significant differentially expressed genes identified using DESeq2 comparing gene expression data in brains of male and female fish from Control and 60% GA*.

Comparison	Gene name	Ensembl ID	Base mean	log2Fold Change	lfcSE (Standard Error)	*p*_adj_	Zebrafish Information Network
Control group only (female VS male)	*ier2a*	*ENSDARG00000099195*	460.0414	1.25	0.233	*p* < 0.01	(http://zfin.org/ZDB-GENE-030131-9126)
Control group only (female VS male)	*egr1*	*ENSDARG00000037421*	4612.752	1.29	0.249	*p* < 0.01	(http://zfin.org/ZDB-GENE-980526-320)
Control group only (female VS male)	*egr4*	*ENSDARG00000077799*	1441.57	1.48	0.312	*p* < 0.05	(http://zfin.org/ZDB-GENE-080204-90)
60% GA VS Control (females only)	*cart1*	*ENSDARG00000070930*	12.66	− 5.66	1.1380	*p* < 0.05	(http://zfin.org/ZDB-GENE-030131-8025)
60% GA VS Control (females only)	*slc1a2a*	*ENSDARG00000052138*	9.29	− 6.35	1.372	*p* < 0.05	(http://zfin.org/ZDB-GENE-100422-11)
60% GA VS Control (all samples without sex as covariate)	*snoRA74*	*ENSDARG00000102120*	0.463	18.48	2.942	*p* < 0.001	Small nucleolar RNA
60% GA VS Control (all samples without sex as covariate)	*cart1*	*ENSDARG00000070930*	7.48	− 4.72	0.896	*p* < 0.01	(http://zfin.org/ZDB-GENE-030131-8025)
60% GA VS Control (all samples without sex as covariate)	*clec3ba*	*ENSDARG00000076541*	118.9782	3.05	0.649	*p* < 0.05	(https://zfin.org/ZDB-GENE-110411-71)

## Discussion

 Nutritional science using animal models recognises that no single animal model perfectly recapitulates human biology nor replicates every nuance of human nutrition. Zebrafish is a pertinent translational model for nutrition, offering valuable cross-species insight^48^. Thus, it complements, but not replace, mammalian models such as mice and rats. The dosages chosen for this study were based on other animal models, including rats (*Rattus* sp.) and Nile tilapia (*Oreochromis niloticus*). In rats, the GA dosages ranged between 4000 mg/kg and 20,000 mg/kg^49^, which is equivalent to a human equivalent dose (HED) based on body surface area (BSA) of 649—3243 mg/kg^50^. This translates to 2 mg of GA/fish/day (i.e. 4000 mg/kg body weight when assuming fish weight = 0.0005 kg) in our 6% GA experiment. In Nile tilapia and other fish species, a ratio of GA ≤ 10% (total food weight) showed beneficial health effects^51–54^, which translates into the HED of 6% GA in zebrafish (i.e. 4000 mg GA/kg body weight) being the equivalent of 280,000 mg GA per day in a 70 kg human adult. Similarly, 60% GA (i.e. 40,000 mg GA/kg) in zebrafish is the equivalent of 2,800,000 mg GA in a 70 kg human adult. However, when applying the BSA normalisation method, the HEDs are around 256 mg/kg in the 6% GA and 2564 mg/kg in the 60% GA experiments, respectively. The K_m_ factor of a 70 kg human adult with a BSA of 1.81 m^2^ is 39^55^. On the other hand, the K_m_ factor of zebrafish is around 2.5 (weight = 0.0005 kg and BSA = 2 × 10^–4^ m^2^; Table S5 in the *SI Appendix*)*.* In summary, the total HED of 256 mg/kg is around 18 g of GA (number of servings = 4 tsp) and around 180 g (number of servings = 36 tsp) of GA for 2564 mg/kg in a 70 kg human adult. Evidently, even the HED of 60% GA is impractical and not realistic. Therefore, results at the higher exposure (60% GA) are unlikely to generalise to typical human intakes, and extrapolation from zebrafish to humans should be made with caution.

Our results demonstrate that GA* supplementation affects female and male zebrafish at multiple levels, spanning from the gut microbiome to metabolic turnover in the gut and brain, and ultimately, overall fitness. The strength of the effects was dosage dependent, where 6% GA* supplementation had positive effects on the gut microbiome in both females and males, with no further implications on reproductive fitness, whereas the effects of 60% GA* supplementation went beyond the microbiome and affected the metabolic rates in the intestines and overall brain activity, potentially at the cost of reduced reproductive fitness in females. The microbiota-gut-brain axis is increasingly recognised to play a key role in organismal health. Changes in the gut microbiota are associated with a range of neurological conditions such as Alzheimer’s, Parkinson’s disease, autism spectrum disorders (ASD), and potentially even mood^56,57^. GA has previously been shown to enhance cognitive abilities and, more recently, to be anti-epileptic in rats^58^. Our results suggest that the substantial changes in gut microbiota in GA*-supplemented fish after just two weeks may have downstream effects and ramifications from gut health to brain function and behaviour. Due to the highly dynamic nature of the gut microbiome, dietary changes and the availability of nutritional substrates influence its composition and architecture. DFs have been thoroughly documented to exhibit gut modulatory effects in humans and animals, which contribute to their positive health benefits^59,60^. In line with previous findings on other DFs, we demonstrated that GA* can favourably shift the Proteobacteria/Fusobacteria (P/F) ratio towards the more beneficial Fusobacteria, and consequently, *Cetobacterium* (Fig. 2A–C and Fig. S2). In zebrafish, a drop in Fusobacteria and a corresponding increase in the P/F ratio is directly linked with gut dysbiosis^61^. In our study, 6% GA* was sufficient to generate beneficial effects on the gut microbiome over just two weeks. Our findings suggest a possible difference in gut microbiome composition according to sex but primarily highlight the sex-specific response to GA* supplementation.

The role of sex in shaping the gut microbiome is contested and inconsistent^62–66^. However, research has shown that sex-specific hormones can alter the gut microbiome composition by influencing the host-microbiome interactions. At the same time, the sex hormones differentially affect the physiology and behaviour of the two sexes, which in turn affects the microbiome^62,67,68^. However, variation in diet has been consistently shown to exert sex-specific changes on the gut microbiome across different species, including the zebrafish^69–71^. In line with the concept of a sex-specific microbiome, we observed that the increase in the relative abundance of *Cetobacterium* was more pronounced in females across both GA* treatments (Fig. S2). However, the higher dosage of 60% GA* only reduced the Observed α-richness in females (Fig. 3A). Since the zebrafish display sexual dimorphism in anatomical, physiological, and behavioural aspects^72–74^. We speculate that this sex-specific effect of GA* on the microbiome is due to diet-host interactions. *Cetobacterium*, a prominent bacterium in the Fusobacteria phylum, is known to produce acetate^40^ which can cross the blood–brain barrier to control appetite and glucose metabolism^40,75^. Acetate, like all SCFAs, is primarily produced in the colon due to microbial fermentation of dietary fibres. In mammals, acetate can be endogenously produced by the liver, albeit to a lesser extent^76,77^. In the zebrafish, the current data are suggestive but still scarce, with almost all papers highlighting and emphasising the role of the gut microbiome in generating acetate as the primary source.

**Fig. 7:**
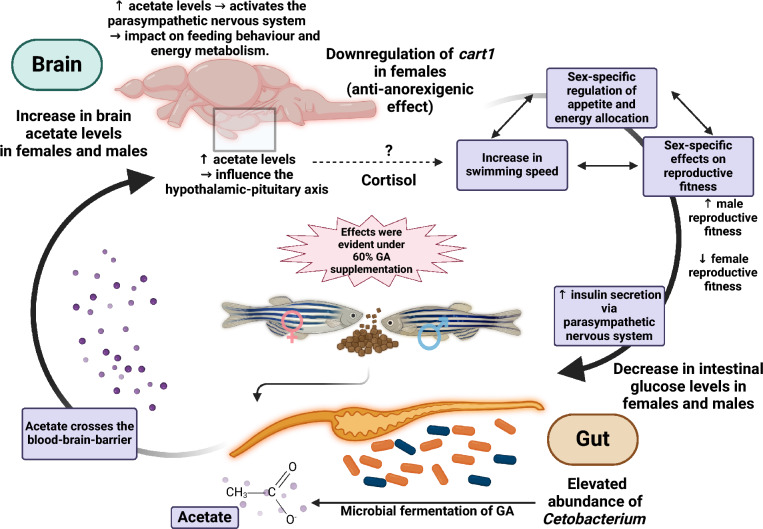
Gum Arabic affects general fitness via the microbiota-gut-brain axis in a sex-specific manner with prominent health ramifications detected under 60% GA supplementation. A schematic model displaying our proposed mechanism of action where ($\uparrow$) denotes an increase, ($\downarrow$) denotes a decrease, ($\to$) denotes a causal effect, ($\leftrightarrow$) denotes a dynamic relation, and (?) denotes a plausible effect. The figure was created in BioRender.com.

Our metabolic profiling analysis revealed abundant levels of glucose in the intestinal tissue of the Control group, in contrast to negligible amounts in the GA*-supplemented fish (Fig. 4). Interestingly, this suggests that GA* can regulate glucose metabolism and appetite through the microbiota-gut-brain axis, particularly via microbial-derived metabolites. Acetate, a key SCFA, modulates immune responses by interacting with immune cell receptors and influencing cytokine production^78^. This modulation, in turn, impacts several metabolic pathways like tryptophan metabolism and its broader health implications include metabolic fitness and cognitive performance^79,80^. We propose that the acetate generated by *Cetobacterium* in response to GA supplementation induced insulin secretion by stimulating the parasympathetic nervous system^40^ (Fig. 7). Subsequently, insulin reduces blood glucose levels by stimulating the glycolytic activity of the fish gut; thus, using up intestinal glucose for metabolism^81^ (Fig. 7). Our metabolomics data do not provide information on insulin levels, most likely because insulin is measured in the plasma and pancreatic tissues using immunoassay techniques^82^. Although insulin was not available in our dataset, tissue glucose was quantified using NMR in both the brain and the intestine. Additionally, acetate plays a role in appetite and feeding behaviour, which subsequently regulates metabolism via interactions with neuropeptides and gut hormones; however, these contrasting effects are dependent on the species, route of administration, and acetate source^75,83–85^. Notably, high levels of acetate were detected in the brain tissues of both females and males under 60% GA* supplementation (Fig. 4). These findings indicate that GA* can support metabolic health and regulate appetite in both sexes.

Our results provide further evidence for a positive effect of GA on the microbiota-gut-brain axis. The observed increase in the relative abundance of *Cetobacterium* under 60% GA* in female zebrafish may be linked to the downregulation of the *cart1* gene, an anorexigenic gene, in female brain tissues via an acetate-dependent pathway. The effects of acetate on the regulation of the *cart1* gene remain inconsistent with some studies indicating no significant change^86^ whilst others show an up-regulation^85^ in gene expression. This discrepancy emphasises that the acetate-mediated effects on metabolism and appetite can differ depending on whether the source is microbial-derived or exogenous^83^. From a mechanistic perspective, our study suggests that the microbial-derived acetate from the GA* fermentation can potentially counter the anorexigenic effects of the *cart1* gene, particularly in females (Fig. 7). Similarly, the gut microbiota has a direct influence on the hypothalamic–pituitary–adrenal axis (HPA), which in turn regulates the organism’s response to metabolic, physiological, and psychological stimuli^87^. Interestingly, there is also a sex-specific link between the *cart1* gene and the HPA axis, particularly in the hypothalamus, with potential downstream effects on the glucocorticoid hormone cortisol and subsequently, stress^88^. In female zebrafish, the observed downregulation of *cart1* may relate to activity in the hypothalamic-pituitary-interrenal (HPI) axis, the teleost homologue of the mammalian HPA axis^89^, with possible ramifications on stress-related behaviours and appetite regulation^90^. However, cortisol levels were not measured, and we can only propose, but not corroborate the exact mechanisms. Since the brain acetate levels were mostly abundant in the females of the 60% GA*-supplemented cohort (Fig. 4), the activation of the HPA axis via acetate might also be dosage-dependent. Furthermore, cortisol and insulin have antagonistic effects on metabolism^91^. We suggest that the stimulation of insulin via the parasympathetic nervous system in response to the microbial-derived acetate has a sex-specific impact on cortisol regulation potentially influencing the contrasting phenotypic data (Fig. 7). The mechanism of the sex-dependent effects of acetate on metabolism and hunger control involves a crosstalk between the microbiota-gut-brain axis and the endocrine system, which warrants further investigation. Additionally, de novo vitamin B_12_ biosynthesis can be derived from *Cetobacterium*^38^ which is essential for maintaining proper brain functioning and supporting the nervous system^92^. Thus, *Cetobacterium*-derived B_12_ can potentially play a role in zebrafish development and influence its locomotion^92–94^. Under 6% and 60% GA*, the raw abundance and relative abundance of *Cetobacterium* was higher in females; however, this apparent increase was only evident in males under 6% GA* supplementation (Fig. 2C and Supplementary Fig. S2). The impact of vitamin B_12_ on reproductive fitness and embryo quality is scarce. From our data, any reproductive fitness under 60% GA* supplementation was primarily evident in male fish and not in females (Fig. 5). After conducting a differential abundance analysis, LEfSe (Linear Discriminant Analysis Effect Size) identified *Cetobacterium* enrichment in females under 60% GA* vs Control (*p* < 0.05); however, effect size was small (LDA score < 2.5). On the other hand, in males, *Cetobacterium* was slightly higher in the Control group compared to the 60% GA* group. However, neither the *p* nor the LDA effect size reached conventional thresholds^95^. For this reason, any contribution of *Cetobacterium*-derived B12, if present, to reproduction or locomotion remains uncertain and warrants further investigation. Separately, DFs have been shown to exhibit immunomodulatory effects on the gut and to shape the homeostasis of the immune system^96,97^. We detected the upregulation of the gene *clec3ba* in 60% GA*-supplemented fish compared to the Control fish (Table 1), which is in line with the idea that GA may have an immunomodulatory effect and increase the expression of C-type lectins in innate immune cells, namely, macrophages/microglia located in the zebrafish brain^98,99^. Incorporating GA into the zebrafish diet can potentially activate defence mechanisms against infections.

Overall health is often directly linked with reproductive fitness^100,101^. We found potentially beneficial effects of GA* supplementation on male reproductive fitness, particularly at high dosages, whereas the effects were detrimental in females. In animals, the implications of DFs on the reproductive parameters of both sexes have been promising. In boars, the incorporation of inulin and wheat bran during the pre-puberty period enhanced testosterone and semen production^102^. Also, in pigs, the incorporation of a variety of DFs primarily before mating, enhanced reproductive fitness and offspring survival by improving oocyte maturation^103^. The health implications of DFs on human reproduction are currently understudied as most research has focused on metabolic health, but a high-fibre diet can reduce the reabsorption of oestradiol in the gut and subsequently be detrimental to the menstrual cycle in women due to the diet’s influence on the hypothalamic-pituitary axis, supporting the potential role of the gut-brain axis in reproduction^104,105^. Moreover, female gonadal hormones can have contrasting effects; therefore, a low intake of DF may negatively affect male fertility^106^. A possible explanation for the positive impact of GA on male reproductive fitness in our study is that it improves sperm quality as previously reported in mammals^24,107,108^. Also, GA can influence the steroid hormones governing gametogenesis^108–110^ and exhibit antioxidant activity to protect the gametes from damage^25,27,107,110^ in both males and females. Despite potentially displaying some adverse effects under specific conditions, DFs play a crucial role in safeguarding metabolic health which in turn can have positive ramifications on fecundity and reproductive nutrition^111,112^. The association between DFs and reproduction is not linear and likely dosage-dependent, which warrants further investigation. Our study highlights the importance of considering differences in female and male physiology when making dietary recommendations and the potential role of the microbiota-gut-brain axis has on reproductive fitness^67,113–115^.

One possible reason for the associations detected between the 60% GA* dosage and reproductive fitness may be the behavioural changes we identified. We observed that fish supplemented with 60% GA* exhibited increased speed (mm/s) but without swimming a longer distance (mm) compared to Control fish (Fig. 6A and B)*.* This is indicative of a burst swimming pattern rather than a sustained movement. We propose three different possible explanations for the increased speed in GA*-supplemented fish. The first theory is that this hyperactivity may benefit males during courtship and spawning activities, where the males rapidly chase females while nudging their flanks with their snouts and attempting to lead them to the spawning site^116^. The second theory relates to foraging and prey-capture behaviour^117,118^, which could be linked to the downregulation of the *cart1* gene (anti-anorexigenic effect) in the 60% GA*-supplemented females and may induce an increased desire for food or a change in feeding behaviour^119–121^ (Table 1). The increase in activity under GA* supplementation could, in turn, explain the reduced reproductive fitness in females caused by their energy allocation strategy, which favours somatic growth over ovarian growth and reproduction^122–124^. Thirdly, since the HPA axis might be involved, this increase in locomotion observed in the GA*-supplemented fish, specifically the rapid burst in swimming (speed) without increased exploration (distance) might be related to the role of cortisol in response to stress or anxiety^125^. Additional studies investigating the possible behavioural and physiological phenotypes of stress and anxiety in zebrafish fed a high-dietary fibre diet are warranted. According to UK guidelines, human adults should aim to consume 30 g of dietary fibre daily for optimal health benefits^126^. On average, most human clinical trials evaluating the effects of GA on different medical diseases provide around 25–30 g GA per day, which is the equivalent of 357—429 mg per kg body weight^22,49^. Assuming an adult human weighs 70 kg, a typical human dosage would be approximately 350 mg/kg which is equivalent to around 5 teaspoons (1 tsp = approx. 5 g) of GA powder per day. Interestingly, the European Food Safety Authority (EFSA) suggests that a numerically Acceptable Daily Intake (ADI) for GA is not required as even oral intake of large amounts (430—760 mg/kg per day) is tolerated and nontoxic; albeit, with potential gastrointestinal discomfort^127^.

## Conclusion

A diet rich in DFs such as GA yields downstream health benefits on the gut microbiome, metabolic, and cognitive functions. Despite that, the optimal daily nutritional requirements are still highly debated^128–130^ and we are just beginning to understand the effects of DFs on overall fitness^131,132^. Additionally, sex differences are often not considered, and varying results of mixed-sex cohorts are typically reported^133,134^. Our results suggest that GA* can positively affect the gut microbiome composition and subsequently influence brain gene expression and functions. However, we also found negative effects on female reproductive fitness at very high dosages. It is essential to understand when and how DFs exhibit optimal benefits and to enhance our understanding of the interplay between nutrition and health. We employed a holistic approach and demonstrated that a change in GA*, even over a short period, has substantial effects on the microbiota-gut-brain axis and even reproduction, but in a dosage-specific manner. However, further in-depth investigation and mechanistic studies are warranted to verify our findings. Finally, we anticipate follow-up studies in mammalian models, considering sex as a biological variable, to be carried out to help establish sex-specific guidelines for optimal dietary fibre intake.

## Materials and methods

### Animal ethics statement

AB wild-type zebrafish were obtained from the European Zebrafish Resource Center (EZRC) in Karlsruhe, Germany. The fish were maintained at a 1:1 sex ratio under standard conditions, in compliance with UK Home Office guidelines, in the Controlled Environment Facility (CEF) at the School of Biological Sciences, University of East Anglia (UEA). Experiments were performed with approval from the UK Home Office under project licence: P0C37E901. The work was thoroughly reviewed; ethical approval was granted (ETH2223-0289) through UEA’s AWERB (Animal Welfare and Ethical Review Body) before being approved by the Home Office. Additionally, all experimental methods were performed in accordance with relevant university guidelines and regulations, as well as the ARRIVE guidelines (https://arriveguidelines.org/).

While mammalian animal models like mice or rats are physiologically closer to humans, zebrafish is a well-established model species for studying human conditions, ranging from neurological diseases to cancer and is also a well-established model in food research^48,135,136^. Sharing > 70% genetic homology with the human genome and all fundamental metabolic pathways with mammals makes them a valuable model for studying these pathways in a species with a lower sentient threshold, as issued by the Home Office (https://www.gov.uk/guidance/research-and-testing-using-animals). Moreover, zebrafish have the advantage of high fecundity, external fertilisation, and rapid development allowing easy monitoring of fitness-related traits in early offspring in a completely non-invasive manner. Also, in vivo experiments using zebrafish benefit from the ability to utilise a larger sample size compared to mice. Finally, the zebrafish model can also provide insights into gut-microbiota-host interactions as it shares structural and functional similarities with the human gut^135–138^.

### Zebrafish husbandry

All the experimental fish used in this study were healthy adult fish (approximately 1 year old). We excluded any fish with visible morphological and behavioural impairment, such as bad posture, surface lesions, an abnormal caudal fin, and impaired swimming behaviour. Eight adult fish were housed in 3.5 L tanks (6% GA experiment) and 16 adult fish in 8 L tanks (60% GA experiment) on a recirculating rack system (ZebTEC rack with Active Blue technology, Tecniplast, UK) at 28 °C and kept on a 14:10 h light: dark cycle. These zebrafish were fed, ad libitum*,* three (weekend) or four (weekdays) times daily, where the standard dry feed (ZEBRAFEED 400–600 by SPAROS, Área Empresarial de Marim, Lote C, 8700–221 Olhão, Portugal) was given once or twice a day, and the live *Artemia franciscana,* from the Great Salt Lake, Utah, USA (Sep-Art Artemia Cysts, Ocean Nutrition) enrichment twice per day. Zebrafish husbandry is not fully standardised, and practices vary among institutions. The current recommendation is to feed adult zebrafish between 3 and 5% of their body weight (split into 1 or 2 meals)^139,140^. We opted for slightly over 3% of body weight per feed ration, as we believe it strikes a good balance between cost efficiency, preservation of good water quality, and proper maintenance of the adult fish. All fish were fed ad libitum from the same batch of standard dry feed, indicated by the lack of active food searching and the presence of residual feed at the bottom of the tanks.

### Formulation of diets and the source of gum Arabic

The control diet consisted of the pre-formulated commercially available ZEBRAFEED 400–600 by SPAROS, which meets the nutritional requirements of zebrafish. The manufacturer reports a crude fibre content of 1.8% for the basal diet (Weende method), which only quantifies insoluble fibre, such as cellulose and lignin, and does not capture soluble DFs derived from GA^141^. The GA* used in the present study was collected in Sudan and taxonomically identified as *Acacia senegal* va*r. senegal* and is from the same batch previously used in a clinical trial as part of The GARDS Study^13^. The Gum Arabic Board of Sudan provided it in spray-dried powder format. We used food-grade gum Arabic (E-414) from *Acacia senegal var. senegal*. Because GA composition, especially protein content linked to emulsifying properties, varies by species and processing, and we did not independently certify this batch against the E-414 specification in Reg. (EU) 231/2012 (https://eur-lex.europa.eu/legal-content/EN/TXT/PDF/?uri=CELEX%3A02012R0231-20210803)^127^, such variability should be considered in replication.

The GA* was fed to the fish by supplementing their dry food. Briefly, the desired amount of GA* was weighed and dissolved in a minute amount of distilled water, enough to allow the GA* to be dissolved entirely then mixed with a weighed amount of standard feed at a specific percentage (6 or 60% w/w), creating a paste that was then spread out to form thin sheets and left to air dry. Using a coffee grinder, the dried sheets were broken down into small ingestible particles, then placed in an incubator at 30 °C overnight until they were fully dry. All diets were kept in opaque and airtight containers in the fridge (4 °C) for the duration of the study. Daily rations were pre-weighed and sealed in labelled Eppendorf tubes, which were also kept in the fridge until use.

### Experimental design

Two independent experiments were conducted for 6% and 60% GA* supplementation, each with its control group. For both experiments, fish were divided into two treatment groups (Control and GA*) and maintained under Control or GA* supplementation conditions for two weeks. For the 6% GA* supplementation experiment, eight fish were housed in each 3.5 L tank at a 1:1 sex ratio (4 females and 4 males, total n = 142). For the 60% GA* supplementation experiment, 16 fish were kept in an 8 L tank at a 1:1 sex ratio (8 females and 8 males, total n = 128). The sex of adult zebrafish can be easily identified visually and morphologically, especially the AB wild-type strain. For all the fish, sex was confirmed at necropsy by inspection of the gonads (ovary or testes). The stocking density was maintained at 4–10 fish per 1 L of water, consistent with current husbandry recommendations^142^. The experiments were divided into blocks, with each block consisting of one Control tank and one GA tank (6% or 60% GA*). We performed nine blocks in the 6% GA* experiment (nine tanks per treatment with eight fish each: 72 fish of which 36 were males and 36 were females) and four blocks (four tanks per treatment with 16 fish each: 64 fish of which 32 were males and 32 were females) in the 60% GA* experiment (Fig. 1). All the fish were randomly selected from population tanks composed of fish from multiple clutches (different parents); sibling matings were not used to establish these tanks. All selected fish were from the same age cohort. Within experimental tanks, fish were randomly allocated to either the Control diet or the GA*-supplemented diet. Additionally, in each block, the tanks were randomly assigned a location on the rack system to minimise confounders. The sample size was determined a priori based on previous pilot experiments conducted within the group. In short, a total sample size of n = 80 fish (40 Control and 40 GA-supplemented) of a 1:1 sex ratio was sufficient to detect between-sex differences in reproductive fitness. In this study, we implemented Reduction (3Rs) and prespecified smaller cohorts without compromising power to detect effects on reproductive fitness/phenotypic data. In the 6% GA* experiment, we used total n = 72 fish (36 Control, 36 GA; 1:1 sex ratio) and in the 60% GA* experiment we used total n = 64 fish (32 Control, 32 GA; 1:1 sex ratio) (Fig. 1).

Our reasons for selecting 6% and 60% GA* are outlined at the start of the Discussion. On a body-weight basis, the zebrafish dosages were intentionally higher than typical human intakes because GA is water-soluble and may leach from feed when delivered in water. Higher inclusion helps ensure actual ingestion of the target amount. In both experiments, the Control fish were under the standard feeding regime of dry and live feed described earlier, whereas the GA fish were fed dry feed supplemented with 6% or 60% GA* and live *Artemia*. To ensure proper nutrition and avoid caloric restriction, all fish were fed ad libitum over 3% of their body weight/day^139,140,143^. In both experiments, the GA*-supplemented diet was fed to the experimental fish while the control fish received the equivalent amount of dry standard feed without GA supplementation. To compensate for any anticipated differences in macronutrients (protein, lipids, and total carbohydrates) between the Control and GA* diets, particularly the 60% GA*, we made additional efforts during feeding. All fish were provided with concentrated *Artemia* nauplii (> 225,000 hatched shrimp per gram) twice a day, during weekdays. *Artemia* is an excellent source of protein (54%) and a significant source of lipids (11%)^144^. Additional effort was made to ensure that the fish under the experimental diets (6% and 60% GA*) received generous amounts of *Artemia* nauplii to meet or even exceed their nutritional requirements^145^. Additionally, we ensured that the water flow was adjusted during *Artemia* feeding for all tanks to prevent rapid flushing. This compensates for any macronutrient difference between the dry diets and mitigates any confounding effects, in addition to the randomisation in fish tank allocation. In support of this, across all reproductive and developmental outcomes examined, macronutrient levels (protein, lipid, and carbohydrates) did not significantly predict variation in clutch production, egg number, fertilisation, or survival outcomes. In contrast, where significant effects were observed, notably for egg number (*p* = 0.001) and number dead at 24 hpf (*p* = 0.01, both under 60% GA* treatment), GA treatment explained more variance and substantially improved model fit, as indicated by lower AIC/BIC (Akaike Information Criterion/Bayesian Information Criterion) values and significant likelihood ratio tests. These results suggest that GA treatment, rather than macronutrient composition, is the primary driver of the observed differences in these reproductive endpoints (Table S6 in the *SI Appendix*). The dry feed was standardised by pre-weighing and then storing in labelled Eppendorf tubes as follows: 0.133 g for eight fish (6% GA*) and 0.266 g for 16 fish (60% GA*). More specifically, an adult fish weighing 500 mg and being fed over 3% of its body weight is the equivalent of just over 15 mg of dry food/fish. In the 6% GA* experiment, we fed 3.325% of 500 mg which is around 16.625 mg of dry feed (8 fish per tank X 16.625 mg = 0.133 g) twice daily resulting in an average of around 2 mg GA per day per fish. Following the same rationale, we fed each fish in the 60% GA* experiment, with an average of approximately 20 mg GA per day.

Reproductive-fitness assessments were conducted under blinded conditions. Following the dietary intervention, each fish was assigned a unique ID, and breeding was conducted without reference to treatment groups (Control or GA*). Spawned embryos were then linked to their parental IDs; however, the allocation key linking IDs to treatment was withheld from those counting the embryos until all quantifications were complete. Thus, the researchers counting the embryos remained blinded. For tissue analyses, samples were randomly selected to ensure balanced representation across treatment groups and experimental blocks.

### Genomic DNA extraction and 16S rRNA sequencing

Genomic DNA was isolated from n = 50 adult zebrafish intestines (6% GA experiment: 14 Control fish and 16 GA fish; 60% GA experiment: 10 Control fish and 10 GA fish) using FastDNA™ SPIN Kit for Soil (MP Biomedical, product code:116560200-CF) as per manufacturer’s recommendations (Fig. 1). This genomic DNA was sent to Source BioScience (UK) in Cambridge (Source Bioscience Sequencing, William James House, Cowley Road, Cambridge, CB4 0WU) for 16S rRNA sequencing (*SI Appendix,* Table S1) with 250 or 300 base pair-end reads. The sequencing platform used was Illumina MiSeq. The bioinformatic microbiome analysis pipeline QIIME2 was used to attain expression data^146^. The R package *phyloseq* 1.44.0^147^ was used for the downstream analysis.

### Metabolites extraction and ^1^H NMR metabolomic analysis

Due to the small amount of tissue in each organ, we pooled brains and intestines from two or three fish per treatment and sex, resulting in six pooled samples per tissue (*SI Appendix,* Table S2). The extraction protocol was conducted and optimised in the Le Gall lab as previously described^148^. High-resolution [^1^H] NMR spectra were recorded on a 600-MHz Bruker Avance spectrometer fitted with a 5-mm TCI proton-optimized triple resonance NMR inverse cryoprobe and a 24-slot autosampler (Bruker, Coventry, England). All identified metabolites (*SI Appendix,* Table S3) were quantified using Chenomx NMR suite 8.6 software (Edmonton, AB, Canada)^149^.

### Reproductive fitness and locomotion assessment

The 6% GA experiment included a total of n = 144 fish (72 Control and 72 GA), whilst the 60% GA experiment included a total of n = 128 fish (64 Control and 64 GA) of equal sex ratio (Fig. 1). We assessed reproductive fitness for both 6% GA fish and 60% GA fish, in addition to their respective control groups.

At the end of the two-week experimental period, natural spawning was initiated by pairing each experimental fish with a non-experimental fish of the opposite sex. The fish were placed in external breeding tanks, separated by a divider, the day before spawning. The breeding tanks were also covered with an opaque cloth to ensure darkness until the next day. On the morning of the experiment**,** after removing the opaque cover and dividers, the breeding tanks were checked hourly for the presence of eggs. In the event of success, the eggs were collected from the tanks and incubated at 28 °C in a 0.01% solution of system water and methylene blue. The resulting clutches were assessed for the different fitness traits at two time points: 2 and 24 hpf (hour post fertilisation).

In the 60% GA experiment, fish were recorded for 60 min in an isolated room whilst being placed in a special tank. All recordings were captured by a GoPro HERO4, and tracking was performed with the idtracker.ai software^150^. The specific settings for the artificial intelligence (AI) tracking are mentioned in the *SI Appendix,* Materials and Methods.

### RNA extraction and RNA-seq

Brain samples from a total of n = 40 fish (20 Control fish and 20 60% GA fish) were collected by dissection following euthanasia by metomidate hydrochloride overdose (Aqua-

calm by Syndel, 4131 Mostar Rd #9, Nanaimo, BC V9T 6A6, Canada), and kept cool on ice before freezing in liquid nitrogen (Fig. 1). RNA extraction was performed using Quick-RNA™ Miniprep Plus Kit with Zymo-Spin™ IIICG Columns (Capped) & Spin-Away™ Filters (Zymo Research, product code: R1057, Cambridge Bioscience, UK) according to manufacturer’s recommendations. Library construction was performed using a NEBNext Ultra II Direction RNA-seq library kit (poly-A selection, 200-300 bp inserts) by the company Novogene (UK) in Cambridge (25 Science Park, Milton, Cambridge, CB4 0FW). Illumina sequencing PE150 was used, resulting in each sample having a minimum of 300 reads and 6G of raw data per sample. The bioinformatics pipeline “nf-core/rnaseq” version: 3.11.1 (https://nf-co.re/rnaseq/3.11.1)^151^ was used to analyse the RNA sequencing data obtained from the zebrafish brain samples using the Ensembl reference genome file (Danio rerio.GRCz11.109). The DEG analysis was performed using the *DESeq2* package^152^. The analysis was stratified into five comparisons according to the diet condition and/or sex by changing the design formula of the *DESeqDataSetFromMatrix* function.

### Statistical analyses

R (version 4.3.1) in R Studio 2023.06.0 Build 421 was used for all the analyses. The Kruskal–Wallis test was used for the α-diversity and statistical significance was determined with a*p*_adj_ < 0.01. Statistical significance in β-diversity was determined using PERMANOVA with a *p* < 0.05. GLMM and LMER models from the library *lme4* were used for the phenotypic and AI data, respectively^153^^154^,. For greater detail regarding these analyses, please refer to the *SI Appendix,* Table S3. Statistical significance for the differentially expressed genes was determined by a log2 fold change with a *p*_adj_< 0.05.

### Preprint

A previous version of this manuscript was published as a preprint 10.1101/2024.10.04.616708.

## Supplementary Information

Supplementary Information.

## Data Availability

The datasets generated and/or analysed during the current study are available in the GEO repository (https://www.ncbi.nlm.nih.gov/geo/) under accession numbers GEO: GSE245252 and GEO: GSE245568. Also, the R scripts used for statistical analysis and graphical representation are available on GitHub (https://github.com/asdfjohnny1/Gum_Arabic_Manuscript). The datasets generated during and/or analysed during the current study are available from the corresponding author on reasonable request.
