# Supplementary material for: Gum Arabic modulates the microbiota-gut-brain axis and affects general fitness in zebrafish
Source: Sci Rep. 2025 Oct 2;15:34465. doi: 10.1038/s41598-025-17665-z (PMC12491494; doi:10.1038/s41598-025-17665-z)
Supplement: Supplementary file 1 — Supplementary Information. [file 41598_2025_17665_MOESM1_ESM.pdf]

Supporting Information for

Gum Arabic modulates the microbiota-gut-brain axis and affects general fitness in zebrafish

Justin Abi Assaf[a*], Jean-Charles de Coriolis[a], Alice May Godden[a], Eve Redhead[a], Jamie Bartram[a], Jayme Cohen-Krais[a], Karina Silova[a], Zoe Crighton[a], Gwenaelle Le Gall[b], Saber Sami[b], Sami Ahmed Khalid[c], and Simone Immler[a*]

[a]School of Biological Sciences, University of East Anglia, Norwich Research Park, NR4 7TJ, Norwich, UK

[b]Norwich Medical School, University of East Anglia, Norwich Research Park, NR4 7TJ, Norwich, UK

[c]Faculty of Pharmacy, University of Science and Technology, Omdurman, Sudan

[*]To whom correspondence should be addressed.

Email: J. Abi Assaf (J.Abi-Assaf@uea.ac.uk), S. Immler (s.immler@uea.ac.uk)

Materials and methods

Genomic DNA extraction (gDNA) and 16S rRNA sequencing

Whole intestines were harvested from euthanised fish by dissection on ice and flash frozen in liquid nitrogen before storage at -80 °C. Before gDNA extraction, frozen samples were homogenized with a handheld homogeniser and pestle and kept on ice. Sequencing libraries were produced with 16S Amplicons V3-V4 by Source Bioscience, Cambridge, UK. The adapter sequences are listed in Table S1. The first ten samples from our first two blocks were sequenced on a MiSeq instrument with 250 base pair (bp) paired-end reads. A PhiX spike-in of 20% was used, and samples were sequenced on one lane on a MiSeq instrument. A further 40 samples were then prepared to produce sequence libraries with IDT for Illumina-Nextera DNA Unique Dual Indexes, using 16S Amplicons V3-V4 and then sequenced in separate sequencing experiment with 300 bp paired-end reads again on a MiSeq (762) instrument. Sequence quality was initially checked with FastQC (https://www.bioinformatics.babraham.ac.uk/projects/fastqc/). Using QIIME2, the sequences were trimmed, and adapters were removed using *cutadapt* in the QIIME2 environment. Samples were demultiplexed and *Dada2* was used to denoise and to trim using the following parameters "--p-trunc-len-f 240 \ --p-trunc-len-r 240 \--p-trim-left-f 13 \--p-trim-left-r 13 \". Feature tables were then generated and visualised before phylogenetic diversity analysis. Taxonomic assignment was performed using the SILVA database release 132 (https://www.arb-silva.de/fileadmin/silva_databases/qiime/Silva_132_release.zip)[1]. Reads were delineated from the database based on the 341 and 806 primers listed in (Table S1). Our *silva* database was then trained using "qiime feature-classifier fit-classifier-naive-bayes". Taxonomy was assigned with qiime *sklearn* and data was exported from the QIIME2 environment to perform the downstream analysis using the package *phyloseq*.

Metabolites extraction and ^{1}H NMR metabolomic analysis

At the end of the 60% GA experimental, we collected intestines and brains from 16 females and 16 males by dissection on ice for further analysis (Table S2). Immediately after dissection, tissues were flash frozen in liquid nitrogen and stored at – 80 °C until further processing. In brief, the samples were weighed and placed in labelled Eppendorf tubes before adding some glass beads to them. Working on ice, 200 µL of ice-cold methanol and 42.5 µL of ultra-pure cold water were added to each tube before vortexing them. The samples were disrupted using

a tissue lyser. After lysis, 100 µL of ice-cold chloroform were added and the tubes were vortexed again before adding another 100 µL of ice-cold chloroform in addition to 100 µL of ultra-pure cold water. The tubes were vortexed one more time and allowed to sit on ice for 15 min, followed by centrifugation at maximum speed for 5 min, creating two visible layers. 250 µL of the top aqueous phase were transferred to new labelled Eppendorf tubes and left to dry gently in an oven for 48 hrs. The bottom chloroform layer was also collected into different new labelled tubes, evaporated, and stored. On the day of the NMR measurement, the aqueous layers were reconstituted in 550 µL of NMR phosphate buffer solution. All samples were then vortexed, and 500 µL was transferred to a 5 mm NMR tube for spectral acquisition.

Phenotypic characterisation and behavioural assessment

To initiate natural spawning, the fish were placed in pairs of opposite sex the day before in breeding tanks and kept separated by a divider. At 7:30 AM the next day, the divider was removed, and an hour of no disturbance was allocated to instigate the breeding in the zebrafish. Irrespective of the spawning outcome, all fish were placed back into their allocated system tanks by midday. The resulting clutches were assessed for various fitness traits including fertilisation success at 2 hpf, survival at 2 and 24 hpf, and the presence or absence of any developmental abnormalities[2] at 2 and 24 hpf. For the AI recording, the fish were placed in a round tank with a diameter of 235 mm and containing 2 L of system water. For the videos, a 40-minute clip was obtained by cropping out the first and last 10 minutes of each one-hour-long recording to reduce biases caused by the new environment and to standardise the footage. They were then further cropped for AI analysis. A total of 40 clips (5 clips x 2 treatments x 4 blocks), each lasting 5 min, were attained before each clip was analysed separately using the default idtracker.ai documentation.

R analysis

The following additional packages were used to generate plots, to handle data, and to perform statistical analysis: *ggplot* 2 3.4[3], *Ape* 5.7-1 PMID: 30016406[4], *Readxl* 1.4.3[5], *dplyr* 1.1.2[6], *Tibble* 3.2.1[7], *Vegan* 2.6-4[8], *ggThemeAssist* 0.1.5[9], *ggpubr* 0.6.0.999[10], *paletteer* 1.5.0[11], *tidyverse* 2.0.0[12], *pairwiseAdonis* 0.4.1[13], *microbiomeMarker* 1.12.2[14], *patchwork* 1.3.1[15]

Supplementary figures

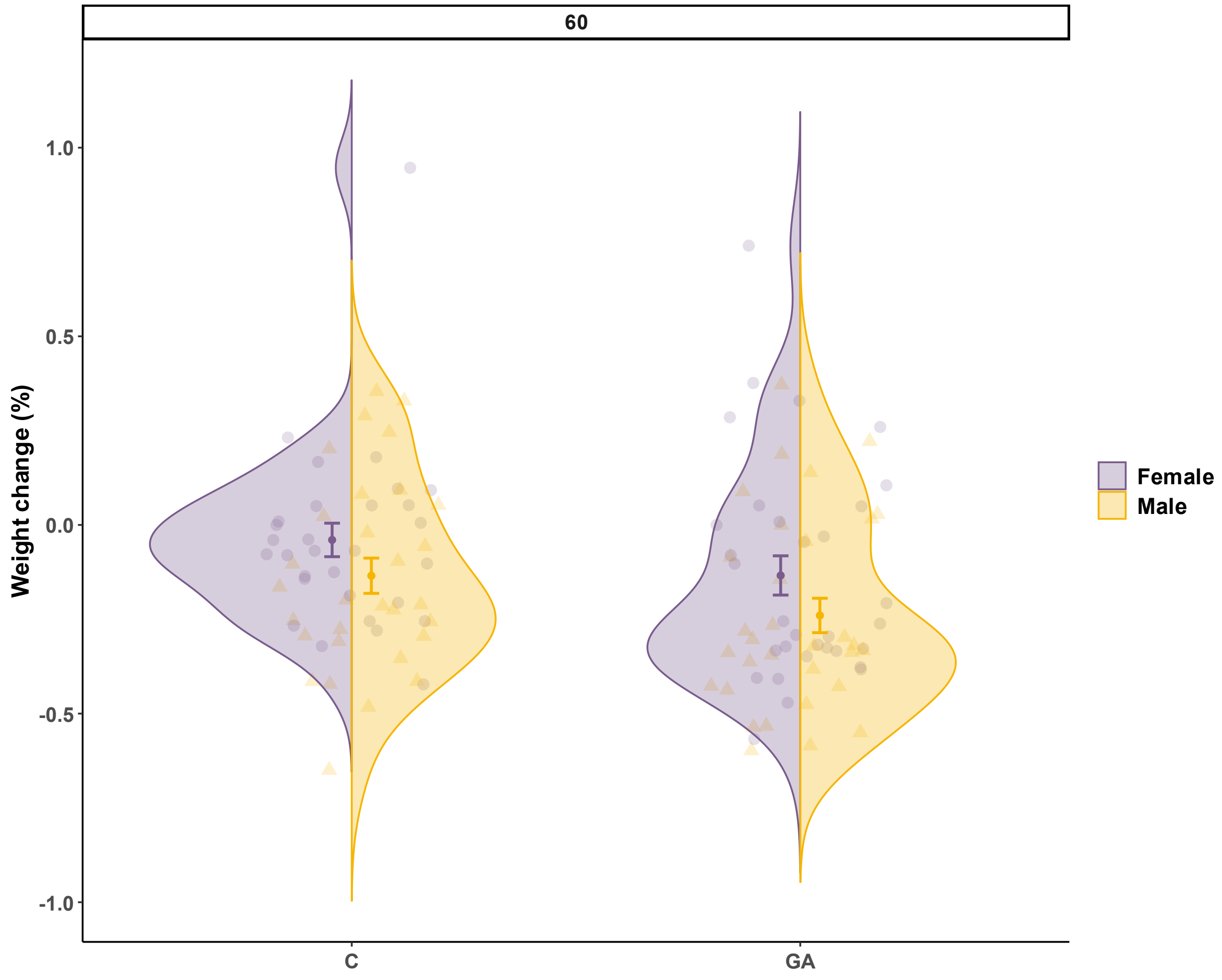

Fig. S1. Female and male fish under 60% GA supplementation showed no statistically significant difference in weight compared to sex-matched controls (linear mixed-effects model, lmer; pairwise EMM contrasts via *emmeans*, Tukey-adjusted *P-value* > 0.05).

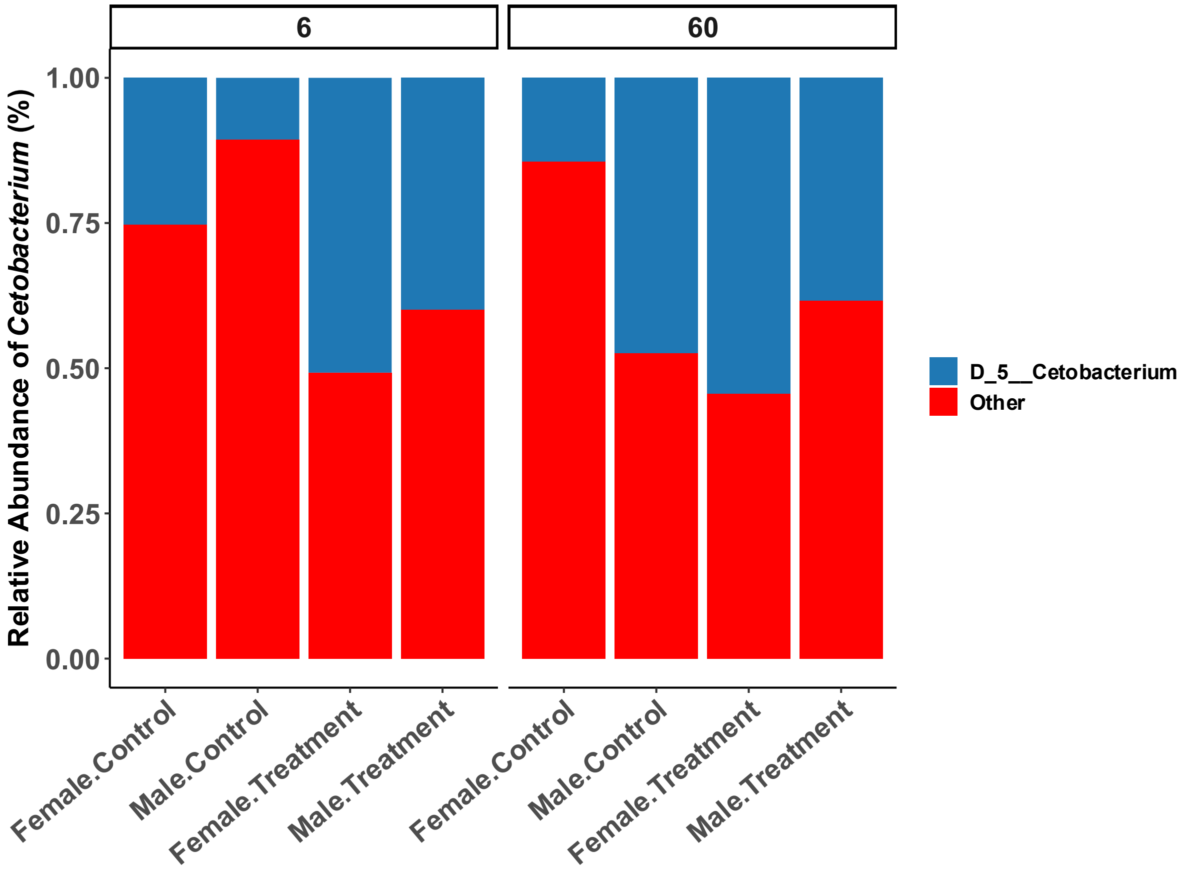

Fig. S2. Under 6% and 60% GA, the relative abundance of *Cetobacterium* was higher in females compared to their control groups; however, this apparent increase was only evident in males under 6% GA supplementation when compared to the control.

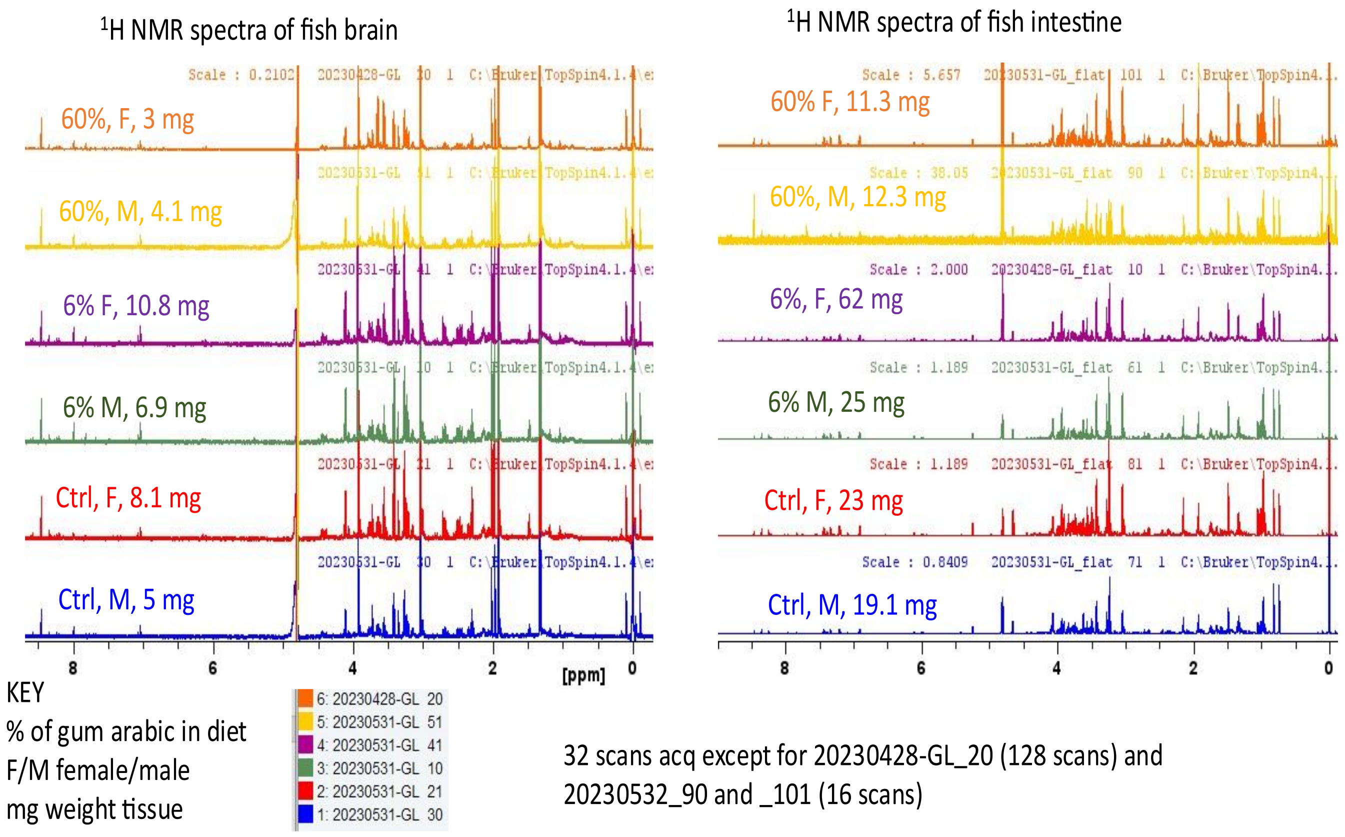

Fig. S3. Raw ^{1}H NMR spectra (brain and intestines). The high field regions are available upon request.

Supplementary tables

Table S1. V3-V4 16S primer sequences and adapter used for library preparation and analyses.

Primer	Sequence (5' -> 3')	Reference
16s amplicon PCR primer forward	TCGTCGGCAGCGTCAGATGTGTATAAGAGACAGCCTACGGGNGGCWGCAG	[16]
16s amplicon PCR primer reverse	GTCTCGTGGGCTCGGAGATGTGTATAAGAGACAGGACTACHVGGGTATCTAATCC	[16]
V3-V4 non overhang primer 341F	CCTACGGGNGGCWGCAG	
V3-V4 non overhang primer 806R	GACTACHVGGGTATCTAATCC	
Forward adapter	CTGTCTCTTATACACATCT	
Reverse adapter	CTGTCTCTTATACACATCT	

Table S2. Supplemental information on the samples used in the metabolomics analysis.

Pooled Sample	Individual Sample ID	Weight (mg)
Control Female Brain	47.1 + 48.1 + C22.1	8.1
Control Female Intestine	47.5 + 48.5 + C22.5	23
Control Male Brain	C23.1 + 75.1	5
Control Male Intestine	C23.5 + 75.5	19.1
6% GA Female Brain	T33.1 + T35.1 + T39.1	10.8
6% GA Female Intestine	T33.5 + T35.5 + T39.5	62
6% GA Male Brain	42.1 + 45.1 +T38.1	6.9
6% GA Male Intestine	42.5 + 45.5 + T38.5	25
60% GA Female Brain	110.1 + 91.1	3
60% GA Female Intestine	110.5 + 91.5	11.3
60% GA Male Brain	84.1 + 90.1 + 106.1	4.1
60% GA Male Intestine	84.5 + 90.5 + 106.5	12.3

Table S3. Supplemental information on the metabolites detected by ^{1}H NMR.

Concentrations (mmol.kg^{-1}) in tissue specimen	BRAIN	BRAIN	BRAIN	BRAIN	BRAIN	BRAIN	INTESTINE	INTESTINE	INTESTINE	INTESTINE	INTESTINE	INTESTINE
Sex	M	F	M	F	M	F	M	F	M	F	M	F
Diet	C	C	6%	6%	60%	60%	C	C	6%	6%	60%	60%
2-Oxoglutarate	0.14	0.56	0.62	0.48	0.66	0.55	0.00	0.00	0.00	0.00	0.00	0.00
4-Aminobutyrate	0.64	2.45	1.88	1.48	1.87	2.29	0.17	0.32	0.19	0.03	0.00	0.45
adenosine phosphate	0.07	0.32	0.28	0.36	0.00	0.04	0.00	0.00	0.00	0.00	0.00	0.00
Acetate	1.79	5.49	6.14	4.01	9.39	12.89	1.81	1.84	1.83	0.62	2.37	3.23
Adenine	0.11	0.31	0.38	0.46	0.84	0.79	0.28	0.30	0.21	0.06	0.00	0.36
Alanine	0.16	0.80	0.72	0.56	0.83	1.19	5.27	6.59	4.10	1.07	0.86	7.09
Arginine	0.00	0.00	0.00	0.00	0.00	0.00	2.65	1.51	2.20	0.57	0.51	3.92
Asparagine	0.00	0.00	0.00	0.00	0.00	0.00	1.55	1.90	1.25	0.41	0.43	2.22
Aspartate	0.13	0.70	1.06	0.70	0.99	0.99	1.57	1.79	1.19	0.44	0.43	1.56
N-acetylaspartylglutamate	4.81	20.32	17.44	12.89	28.71	22.20	0.00	0.00	0.00	0.00	0.00	0.00
Betaine	0.13	0.42	0.44	0.49	0.65	0.77	0.36	0.34	0.15	0.03	0.05	0.52
Choline	0.04	0.21	0.20	0.17	0.25	0.46	0.71	0.40	0.39	0.11	0.00	0.74
Citrulline	0.00	0.00	0.00	0.00	0.00	0.00	0.47	0.65	0.58	0.35	0.00	1.51
Creatine	1.65	6.39	6.92	6.23	7.30	9.72	2.17	2.90	2.53	0.71	0.18	2.92
Dimethylamine	0.03	0.23	0.13	0.25	0.19	0.02	0.25	0.24	0.30	0.15	0.00	0.50
Ethanol	0.14	0.39	0.36	0.27	0.66	0.64	0.00	0.00	0.00	0.00	0.00	0.00
Formate	0.98	2.41	3.32	2.08	6.35	7.17	1.12	1.07	0.78	0.33	2.04	1.90
Fumarate	0.00	0.00	0.00	0.00	0.00	0.00	0.05	0.06	0.03	0.02	0.00	0.07
Glucose	0.00	0.00	0.00	0.00	0.00	0.00	14.30	15.59	6.10	1.53	1.05	7.73
Glutamate	0.59	2.06	2.29	1.79	2.79	2.33	2.80	3.56	2.91	0.78	0.14	4.01
Glutamine	0.36	1.46	1.66	1.69	2.15	2.11	2.85	2.42	2.17	0.67	0.39	4.16
Glutathione	0.00	0.00	0.00	0.00	0.00	0.00	0.26	0.22	0.24	0.05	0.00	0.31
Glycerol	0.35	1.14	1.30	1.09	1.65	12.36	1.83	2.99	2.10	0.60	0.76	1.37
Glycine	0.19	1.06	1.01	0.73	1.16	1.43	1.96	2.33	1.36	0.59	0.55	2.59
Guanosine	0.00	0.00	0.00	0.00	0.00	0.00	0.18	0.18	0.12	0.03	0.00	0.15
Histidine	0.30	0.76	1.43	0.64	1.47	2.51	0.85	1.11	0.64	0.17	0.00	0.41

Inosine phosphate	0.00	0.00	0.00	0.00	0.00	0.00	0.11	0.10	0.13	0.10	0.00	0.13
Inosine	0.11	0.21	0.31	0.36	0.34	0.95	1.23	1.13	0.64	0.20	0.21	1.09
Isoleucine	0.03	0.06	0.09	0.12	0.11	0.26	2.34	2.86	1.80	0.44	0.39	2.93
Lactate	1.62	6.75	11.22	6.74	8.65	11.04	1.97	2.09	1.67	0.45	0.28	2.54
Leucine	0.03	0.06	0.09	0.15	0.18	0.37	4.92	6.84	4.52	1.10	0.82	7.39
Lysine	0.00	0.00	0.00	0.00	0.00	0.00	5.95	6.94	4.05	0.85	0.33	7.67
Maltose	0.00	0.00	0.00	0.00	0.00	0.00	0.98	0.34	0.00	0.09	0.00	0.00
Methanol	0.21	0.60	0.68	0.40	1.18	1.61	0.29	0.23	0.16	0.06	0.29	0.37
Methionine	0.03	0.09	0.09	0.07	0.17	0.11	1.62	2.21	1.45	0.29	0.17	2.34
N-Acetylcysteine	0.00	0.00	0.00	0.00	0.00	0.00	0.36	0.21	0.22	0.08	0.00	0.32
Niacinamide	0.00	0.00	0.00	0.00	0.00	0.00	0.15	0.21	0.11	0.11	0.00	0.31
N-Acetylaspartate	0.55	3.06	2.92	2.15	3.09	3.10	0.00	0.00	0.00	0.00	0.00	0.00
O-Acetylcarnitine	0.01	0.03	0.04	0.02	0.04	0.06	0.02	0.01	0.08	0.02	0.00	0.04
O-Acetylcholine	0.01	0.03	0.04	0.02	0.04	0.06	0.00	0.00	0.00	0.00	0.00	0.00
O-Phosphocholine	0.03	0.14	0.13	0.11	0.19	0.18	2.21	0.79	0.86	0.27	0.16	1.88
Ornithine	0.00	0.00	0.00	0.00	0.00	0.00	0.07	0.30	0.41	0.04	0.00	0.20
Phenylalanine	0.00	0.00	0.00	0.00	0.00	0.00	2.72	3.07	1.67	0.37	0.28	2.43
Proline	0.00	0.00	0.00	0.00	0.00	0.00	1.67	2.07	1.54	0.44	0.50	2.51
Serine	0.10	0.27	0.24	0.33	0.34	0.40	2.98	3.73	3.35	0.92	0.22	4.92
Succinate	0.01	0.01	0.03	0.05	0.07	0.07	0.05	0.08	0.06	0.01	0.00	0.05
Sucrose	0.00	0.00	0.00	0.00	0.00	0.00	0.35	0.00	0.00	0.00	0.00	0.00
Taurine	1.33	6.28	6.61	6.18	6.05	9.09	10.24	8.28	9.09	2.09	1.33	8.57
Threonine	0.00	0.00	0.00	0.00	0.00	0.00	1.68	2.08	1.81	0.52	0.20	2.97
Tryptophan	0.00	0.00	0.00	0.00	0.00	0.00	0.47	0.64	0.37	0.10	0.00	0.53
Tyrosine	0.00	0.00	0.00	0.00	0.00	0.00	2.70	3.06	2.16	0.41	0.41	4.46
UDP-galactose	0.00	0.00	0.00	0.00	0.00	0.00	0.37	0.19	0.24	0.07	0.00	0.25
UDP-glucose	0.00	0.00	0.00	0.00	0.00	0.00	0.79	0.33	0.39	0.14	0.00	0.50
Uracil	0.00	0.00	0.00	0.00	0.00	0.00	0.32	0.17	0.24	0.05	0.00	0.50
Uridine	0.00	0.00	0.00	0.00	0.00	0.00	0.11	0.12	0.09	0.02	0.00	0.19
Valine	0.03	0.09	0.07	0.12	0.19	0.28	3.21	3.75	2.48	0.67	0.44	4.24
Xanthine	0.64	0.34	2.87	0.94	4.11	3.69	0.00	0.17	0.16	0.35	0.66	0.24
Citrate	0.03	0.14	0.17	0.18	0.11	0.24	0.13	0.10	0.11	0.02	0.00	0.17
Malate	0.11	0.31	0.50	0.42	1.06	1.03	0.82	0.51	0.48	0.15	0.00	0.73
myo-Inositol	0.20	1.05	0.97	0.76	1.38	1.05	0.00	0.00	0.00	0.00	0.00	0.00
sn-Glycero-3-phosphocholine	0.07	0.28	0.50	0.31	0.56	0.66	1.09	4.28	1.77	0.19	0.00	2.32

Table S4. Supplemental information on the statistical models.

	Clutch production (6% GA)				Clutch production (60% GA)			
**Effect**	**Est**	x^2	**DF**	***P***	**Est**	x^2	**DF**	***P***
GA treatment	0.36	0.40	1	0.52	-0.41	0.54	1	0.46
Sex	0.31	0.35	1	0.55	-0.27	0.27	1	0.61
Treatment: Sex	-0.49	0.47	1	0.49	1.32	3.18	1	0.075
	**Random effects variance: Tank: 0.23, SD=0.48**				**Random effects variance: Tank: 0.05, SD= 0.24**			

	Total egg number (6% GA)				Total egg number (60% GA)			
**Effect**	**Est**	x^2	**DF**	***P***	**Est**	x^2	**DF**	***P***
GA treatment	-0.19	0.10	1	0.76	0.34	0.13	1	0.72
Sex	-0.16	0.02	1	0.88	0.26	0.75	1	0.39
Treatment: Sex	0.22	0.22	1	0.64	0.37	12.56	1	< 0.001 ***
	**Random effects variance: Observation: 0.74, SD = 0.86 Tank: <0.001, SD = <0.001 2h counter: 0.03, SD = 0.16**				**Random effects variance: Observation: 0.35, SD = 0.60 Tank: 0.06, SD = 0.24 2h counter: 0.15, SD = 0.39**			

	Unfertilised embryos (6% GA)				Unfertilised embryos (60% GA)			
**Effect**	**Est**	x^2	**DF**	***P***	**Est**	x^2	**DF**	***P***
GA treatment	-0.27	0.08	1	0.77	1.23	0.39	1	0.53
Sex	0.19	0.26	1	0.61	-0.14	4.57	1	0.03 *
Treatment: Sex	0.21	0.03	1	0.86	-1.77	2.92	1	0.09
	**Random effects variance: ID: 3.3, SD = 1.82 2h counter: 0.21, SD = 0.46**				**Random effects variance: ID: 1.49, SD = 1.2 2h counter: 1.15, SD = 1.7**			

	Dead embryos 24h (6% GA)				Dead embryos 24h (60% GA)			
**Effect**	**Est**	***x^2***	**DF**	***P***	**Est**	***x^2***	**DF**	***P***
GA treatment	-0.88	0.55	1	0.46	0.64	0.82	1	0.37
Sex	-1.02	1.54	1	0.22	0.57	1.94	1	0.16
Treatment: Sex	1.02	0.1.77	1	0.18	0.80	6.31	1	0.01 *
	**Random effects variance:<br>Observation: 1.6, SD = 1.3<br>Tank: 0.23, SD = 0.5**				**Random effects variance:<br>Observation: 1.5, SD = 1.2<br>Tank: 0.06, SD = 0.2<br>2h Counter: 0.10, SD = 0.33**			

Table S5. Supplemental information for body surface area (BSA).

Fish	Strain	Sex	Body Surface Area*	Unit
1	AB WT	Female	2.69E+08	μm^2
2	AB WT	Female	2.09E+08	μm^2
3	AB WT	Female	2.09E+08	μm^2
4	AB WT	Male	2.33E+08	μm^2
5	AB WT	Male	2.47E+08	μm^2
6	AB WT	Male	2.41E+08	μm^2

*The body surface area of zebrafish is estimated to be at least 10^{-4} m^2. We took images of the whole fish and used ImageJ to assess the BSA. The raw images can be provided upon request.

Table S6. Supplemental information on the statistical model for macronutrient differences (Control vs 60% GA).

GA	**Outcome**	**Model**	**AIC**	**BIC**	**Chisq**	**df**	***P-value***
6	Clutch Production	Sex + Fat + Protein + Carbs	193.4	205.1	-	-	-
6	Clutch Production	GA_Treatment * Sex Model	194.9	209.6	0.47	1	0.493
60	Clutch Production	Sex + Fat + Protein + Carbs	190.5	-85.6	-	-	-
60	Clutch Production	GA_Treatment * Sex Model	192.2	-83.9	3.2	1	0.07
60	Weight Difference	Sex + Fat + Protein + Carbs	-199.5	104.7	-	-	-
60	Weight Difference	GA_Treatment * Sex Model	198	105	0.5	1	0.47
6	Unfertilised 2hpf	Sex + Fat + Protein + Carbs	297.8	142.9			
6	Unfertilised 2hpf	GA_Treatment * Sex Model	299.8	313.7	0.0326	1	0.86
60	Unfertilised 2hpf	Sex + Fat + Protein + Carbs	181.7	-84.9			
60	Unfertilised 2hpf	GA_Treatment * Sex Model	180.9	-83.5	2.8	1	0.09
6	Egg Number	Sex + Fat + Protein + Carbs	657.5	669.5			
6	Egg Number	GA_Treatment * Sex Model	659.3	673.3	0.2	1	0.6
60	Egg Number	Sex + Fat + Protein + Carbs	555.5	567			
60	Egg Number	GA_Treatment * Sex Model	547.1	560.5	10.4	1	**0.001**
6	Abnormal rate 2hpf	Sex + Fat + Protein + Carbs	124.2	129.9			
6	Abnormal rate 2hpf	GA_Treatment * Sex Model	123.8	130.6	2.4	1	0.1
60	Abnormal rate 2hpf	Sex + Fat + Protein + Carbs	121.5	131.1			
60	Abnormal rate 2hpf	GA_Treatment * Sex Model	123.5	135	0.05	1	0.8
6	Number Dead 2hpf	Sex + Fat + Protein + Carbs	91.9	98.7			
6	Number Dead 2hpf	GA_Treatment * Sex Model	93.8	101.7	0.1	1	0.7
60	Number Dead 2hpf	Sex + Fat + Protein + Carbs	118.7	130.2			
60	Number Dead 2hpf	GA_Treatment * Sex Model	119.1	132.5	1.6	1	0.2
6	Abnormal rate 24hpf	Sex + Fat + Protein + Carbs	216	227.9			
6	Abnormal rate 24hpf	GA_Treatment * Sex Model	218	231.9	0.002	1	0.9
60	Abnormal rate 24hpf	Sex + Fat + Protein + Carbs	180.3	-84.1			
60	Abnormal rate 24hpf	GA_Treatment * Sex Model	180.9	-83.5	1.4	1	0.2
6	Number Dead 24hpf	Sex + Fat + Protein + Carbs	425.8	437.7			
6	Number Dead 24hpf	GA_Treatment * Sex Model	426.1	434	1.7	1	0.2
60	Number Dead 24hpf	Sex + Fat + Protein + Carbs	370.2	381.7			
60	Number Dead 24hpf	GA_Treatment * Sex Model	366.1	379.5	6.1	1	**0.01**

References

1. Quast, C. *et al.* The SILVA ribosomal RNA gene database project: Improved data processing and web-based tools. *Nucleic Acids Res* **41**, (2013).
2. Kimmel, C. B., Ballard, W. W., Kimmel, S. R., Ullmann, B. & Schilling, T. F. Stages of embryonic development of the zebrafish. *Developmental Dynamics* **203**, 253–310 (1995).
3. Wickham, H. Data Analysis. in *ggplot2: Elegant Graphics for Data Analysis* 189–201 (Springer International Publishing, Cham, 2016). doi:10.1007/978-3-319-24277-4_9.
4. Paradis, E. & Schliep, K. Ape 5.0: An environment for modern phylogenetics and evolutionary analyses in R. *Bioinformatics* **35**, 526–528 (2019).
5. Wickham, H. & Bryan, J. readxl: Read Excel Files. https://readxl.tidyverse.org (2023).
6. Wickham, H., François, R., Henry, L., Müller, K. & Vaughan, D. dplyr: A Grammar of Data Manipulation. https://dplyr.tidyverse.org (2023).
7. Müller, K. & Wickham, H. tibble: Simple Data Frames. https://tibble.tidyverse.org/ (2023).
8. Oksanen, J. *et al.* vegan: Community Ecology Package. https://vegandevs.github.io/vegan/ (2024).
9. Gross, C. & Ottolinger, P. ggThemeAssist: Add-in to Customize ‘ggplot2’ Themes. https://github.com/calligross/ggthemeassist (2016).
10. Kassambara, A. ggpubr: ‘ggplot2’ Based Publication Ready Plots. https://rpkgs.datanovia.com/ggpubr/ (2023).
11. Hvitfeldt, E. paletteer: Comprehensive Collection of Color Palettes. https://github.com/EmilHvitfeldt/paletteer (2021).
12. Wickham, H. *et al.* Welcome to the Tidyverse. *J Open Source Softw* **4**, 1686 (2019).
13. Martinez Arbizu, P. Pairwiseadonis: Pairwise multilevel comparison using Adonis. . https://github.com/pmartinezarbizu/pairwiseAdonis (2020).
14. Cao, Y. *et al.* microbiomeMarker: an R/Bioconductor package for microbiome marker identification and visualization. *Bioinformatics* **38**, 4027–4029 (2022).
15. Pedersen, T. L. patchwork: The Composer of Plots (R package version 1.3.1). https://patchwork.data-imaginist.com/ (2025).
16. Klindworth, A. *et al.* Evaluation of general 16S ribosomal RNA gene PCR primers for classical and next-generation sequencing-based diversity studies. *Nucleic Acids Res* **41**, (2013).
